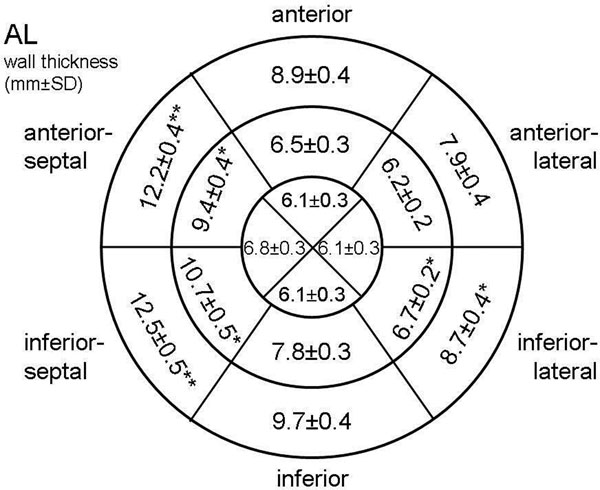# Thickness of the midventricular left ventricular wall is predictive of survival in different forms of cardiac amyloidosis

**DOI:** 10.1186/1532-429X-15-S1-P93

**Published:** 2013-01-30

**Authors:** Fabian aus dem Siepen, Rebekka Kammerer, Katrin A Scherer, Ralf Bauer, Stefan E Hardt, Evangelos Giannitsis, Sebastian Buss, Arnt V Kristen

**Affiliations:** 1 Cardiology, University of Heidelberg, Heidelberg, Germany

## Background

Systemic amyloidosis is a disorder characterized by extracellular deposition of different insoluble protein fibrils in various organs leading to organ dysfunction. Cardiac involvement is associated with limited survival. We aimed to use cardiac magnetic resonance imaging (CMR) to identify structural and functional alterations related to the different forms of amyloidosis, e. g. hereditary and wild-type transthyretin (TTR) amyloidosis as well as light-chain (AL) amyloidosis.

## Methods

In total, 130 patients (82 male, 38 female; mean age 60.7±1.1 years) with different forms of amyloidosis (AL n= 72, TTR n=58) were evaluated by two blinded experienced observers employing a Vector-ECG gated 1.5T whole-body CMR (Achieva Intera^®^ Philips Medical Systems, Best, The Netherlands). The study included SSFP and gadolinium contrast delayed enhancement (CE-CMR) 2-,3-,4-chamber and short-axis planes. EDV, ESV, EF and myocardial mass were analyzed on a standard workstation (Philips Viewform). Regional wall thickness was analyzed in modified 16 segment AHA-model of the left ventricle as well as the interatrial wall and compared between the different forms of amyloidosis. Univariate and multivariate analysis were performed to define predictors of survival.

## Results

**Figure 1:**
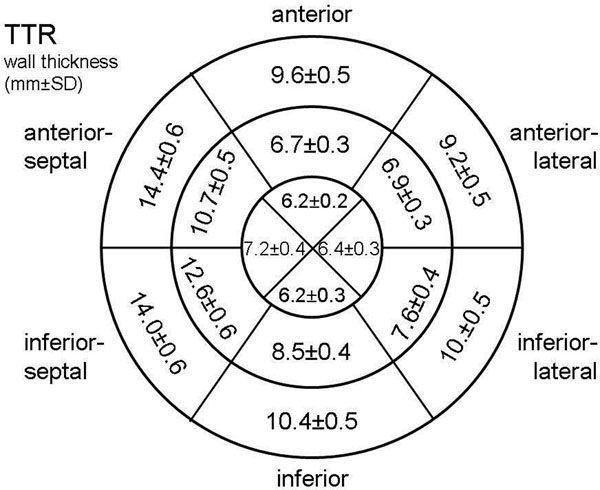


Patients with TTR amyloidosis were significantly older as compared to patients with AL. They had higher left ventricular mass (171.9±8.1g vs. 146.1±6.9g, p<0.01) and thickness of interatrial septum (7.3±0.3mm vs. 5.7±0.2mm; p<0.001) with highest LV mass and thickness of interatrial septum in patients with wild-type amyloidosis (n=26). Distribution of regional wall thickness is shown in figure 1. In AL patients univariate analysis revealed median of maximal wall thickness of the basal (14 mm), midventricular (10 mm) and apical (7 mm) segments as predictors of survival. In multivariate analysis midventricular maximal wall thickness was the only independent predictor of survival. In TTR patients univariate analysis revealed median of maximal wall thickness of the midventricular (13.5 mm) and apical (8 mm), but not basal (16.0 mm) segments as predictors of survival. There was no independent predictor of survival in multivariate analysis of these patients.

## Conclusions

According to this analysis of a large cohort of patients with different forms of cardiac amyloidosis maximal thickness of LV wall in the midventricular segments appears to be predictive of survival in patients with amyloidosis. Further studies are needed to confirm these results in a larger independent patient cohort especially of the TTR type.

## Funding

none

**Figure 2:**